# Women’s experiences of accessing individualized disability supports: gender inequality and Australia’s National Disability Insurance Scheme

**DOI:** 10.1186/s12939-021-01571-7

**Published:** 2021-11-08

**Authors:** Sophie Yates, Gemma Carey, Jen Hargrave, Eleanor Malbon, Celia Green

**Affiliations:** 1 Public Service Research Group, School of Business, UNSW Canberra, Canberra, Australia; 2 Centre for Social Impact, UNSW, Sydney, Australia

**Keywords:** Disability services, Gender equality, Personalized budgets, Individualized funding schemes

## Abstract

**Background:**

Care services in industrialized nations are increasingly moving towards individualized funding models, which aim to increase individuals’ flexibility, choice and control over their services and supports. Recent research suggests that such schemes have the potential to exacerbate inequalities, however none has explored gendered dimensions of inequality. The Australian National Disability Insurance Scheme (NDIS) is a major individualized funding reform, and has a female participation rate of only 37%, despite women and girls making up half of the disability population.

**Methods:**

The objective of the study is to explore possible gendered barriers to applying for and receiving adequate support through the NDIS, and to suggest directions for future research. We report on semi-structured interviews with 30 women with disability and explore their experiences with the NDIS and their perspectives on challenges associated with being a woman seeking disability support in Australia. We analyse the results using thematic analysis.

**Results:**

Most women in our sample reported differences between the experiences of men and women seeking disability support in Australia. Commonly reported gendered barriers to women being able to access the right supports for their disability involve a) confidence, negotiation and self-advocacy, b) gendered discrimination in diagnosis and the medical system, which has implications for disability support access, and c) support for and recognition of caring roles.

**Conclusions:**

These results suggest that women are not receiving equitable treatment with regard to the NDIS, and that further research and policy reform are needed to ensure that women with disability are not further disadvantaged as a result of the move toward individualized funding models.

## Introduction

Disability and aged care services in industrialized nations are increasingly moving towards individualized and personalized funding models, which aim to increase individuals’ flexibility, choice and control over the services and supports that best suit their needs [1]. Although evidence suggests individualized funding schemes have led to benefits for users, including increased satisfaction, a reduction in unmet needs, stronger continuity of care, and more efficient use of scarce resources (e.g. [2, 3]), some have argued that this evidence has originated from those largely in favour of the concept [4] and that whether individualized approaches have consistently created improved outcomes in people’s lives is still very much a matter of debate [5, 6]. One critical issue that has received little attention in the literature is whether individualized funding schemes may benefit different social groups over others.

Early evidence from the Australian National Disability Insurance Scheme (NDIS) suggests that the shift to an individualized funding model for disability appears to be widening some inequalities [7]. At present, the NDIS has a female participation rate of 37%, while ABS data indicate that girls and women form 49% of the disability population in the NDIS-eligible age group of under 65 [8, 9]. This is consistent with data from other liberal democracies, where men and women have similar rates of disability. Women and girls’ underrepresentation in the NDIS has remained steady since the scheme was launched in 2013 [9]. Low female participation in the NDIS is concerning given Australia’s obligations under the UN *Convention on the Rights of Persons with Disabilities*. An objective of the NDIS is to respond to Australia’s obligations under this Convention [10], which emphasizes that women and girls with disability are subject to multiple discriminations, and that signatory states must take measures to “ensure the full development, advancement and empowerment of women” [11].

For those women who do have NDIS access, research from the NDIS trial sites, and more broadly the health literature of women and social and health services, indicates that their needs are less likely than men to be met [12]. While the disability advocacy sector has identified service gaps in relation to women with disability and called for an NDIS Gender Strategy [13], there has been no published research examining gendered experiences of the NDIS, and there is no gender strategy in place to address the low female participation rate.

There are several potential reasons for women’s under-servicing in the NDIS. These may include the fact that women are underdiagnosed in relation to several types of disabilities, particularly those that are most likely to be funded under the NDIS, such as autism spectrum disorder [14]. Women are also more likely to be diagnosed with disabilities that are difficult to get NDIS funding for, such as autoimmune disorders [15] myalgic encephalomyelitis/chronic fatigue syndrome (ME/CFS) [16], and fibromyalgia [17]. Secondly, women and girls are socialized to undervalue and underpromote their own needs and requirements in situations where negotiation is required [18]. This may lead to women being less effective self-advocates than men in schemes such as the NDIS that put an unprecedented emphasis on individuals to navigate care systems and advocate for their own needs and rights [19]. Thirdly, women are more likely to experience complications related to caring responsibilities (particularly mothering) and having those recognized and supported through disability services [20].

This article draws on data collected in an exploratory study about the experiences of women seeking disability support through the NDIS. The objectives were to identify possible gendered barriers to applying for and receiving adequate support through the NDIS, and to suggest directions for future larger-scale research. We interviewed 30 women with disability about their decision to apply for NDIS support (or not), their experiences with the scheme if they were NDIS participants, and whether they felt there were any differences between men and women seeking disability support in Australia. We found that most participants did perceive gendered differences, and many described how these barriers impacted on their ability to get the right supports for their disabilities. Consequently, we argue that the NDIS needs an explicit gender strategy to address inequalities.

We begin by describing the NDIS, and then explore literature relevant to gender inequality and individualized funding schemes. We then describe the methods we used in this study, and present our results, focusing on the three themes identified above. Lastly, we discuss the implications of our findings for policy and practice.

## Background

### The National Disability Insurance Scheme

The NDIS was passed with bipartisan commitment in legislation in 2013, after a significant community campaign which leveraged human rights discourse [21]. Under the NDIS, approximately 500,000 individuals who have a significant and permanent disability will receive personalized funding budgets (just under 10% of Australia’s 4.4 million people with disability) [22, 23]. From these budgets they purchase services and supports that meet their needs – with the aim of giving greater choice and control [22, 23].

It is worth noting though the NDIS launched in 2013, it is still very much in active implementation [1]. This implementation has been characterized by a host of challenges and disruptors which, while not the focus of this paper, impact on participants’ experiences of the scheme [24–26].

### Gender, disability and individualized funding schemes

In this article, we define gender as a series of processes and institutions that distribute power differentially according to a person’s social assignment to the predominant category of male or female [27]. This means that we see gender inequality and the processes that create it as products of socially reproduced oppression rather than innate or essential differences between men and women. Similarly, in adopting the social model of disability, we see impairments as disabling for individuals due to socially imposed barriers such as inadequate transportation, unsuitable infrastructure, and discriminatory attitudes [28].

Following Risman’s (2004) theory of gender as a social structure, we note that gender processes occur at the individual, interactional and institutional levels to produce inequality, and that gender intersects with other oppressive social structures such as race and class to produce different experiences of inequality for different groups of people. However, scholars have cautioned against an ‘additive’ approach that sees the effects of social structures such as disability and gender as having independent effects on a person’s lived experience, adding to or multiplying their disadvantage. Although the impacts of gender and disability may “add up” over time and increase in intensity, as Traustadóttir [29] notes, an additive model will overlook the interactions that occur between them, as well as the distinct ways different configurations of gender and disability result in differing experiences for individuals and groups (see also [30]).

Western welfare states have undergone significant changes in recent decades with the rise of individualized funding models for the provision of care services. Yet despite the foundational feminist literature on welfare states, there has been little, if any, gendered examination of individualized funding schemes. Recent research reveals that the design and administration of these schemes has the potential to entrench or even exacerbate existing inequalities, with a number of demographic factors such as race, age, disability type, and socioeconomic status being highlighted as a significant factors contributing to inequalities [7, 31]. Although gender has been mentioned as another factor contributing to inequality for users of individualized funding schemes such as the NDIS, this has not been explored in the literature and is often mentioned only incidentally (e.g. [7, 32]). Here we draw on feminist and disability literature to highlight potential gendered issues with individualized funding schemes.

#### The ‘problem’ of gender in health and disability

Research is increasingly recognising the way that medical diagnosis, treatment and research has long been biased against women and girls [33, 34]. This is likely to play a role in women’s underrepresentation in the NDIS, given that only certain conditions and levels of impairment are funded under the scheme. While eligibility for the scheme is based on an assessment of functional capacity rather than strictly diagnosis, somewhat in line with the social model of disability, it does rely on medical documentation to ascertain eligibility. For example, more boys and men are diagnosed with autism than women and girls. However, recent research has shown the differential levels of diagnoses likely arise from under-diagnosis in girls and women (e.g. [14, 35, 36]). Gendered socialisation of girls and boys, particularly those at the ‘higher-functioning’ end of the autism spectrum (in contrast to those who are non-verbal or significantly cognitively impaired), may lead to behaviours that camouflage or mask autism in girls but not boys [35]. As Zener [36] points out, these missed diagnoses can create significant mental health impacts for women when they are seeking acknowledgement of social difficulties. Missed diagnoses may also present a problem for women’s access to disability funding schemes such as the NDIS.

Beyond autism, there is also a wide literature on gender bias in medical diagnosis and treatment more generally. Research has shown that for a wide variety of conditions, men receive more extensive investigation and treatment than women [37, 38]. These gender biases also have implications for conditions that have a greater prevalence in women than men, such as ME/CFS and fibromyalgia. Briones-Vozmediano et al. [16] argue that women presenting with ME/CFS are often perceived as ‘complaining’ by health professionals, and the fact that this disease affects women more than men has influenced professional practice regarding whether it is recognized as a severe condition. Given the gender bias in the diagnosis and treatment of pain in women [37], it may be harder for them to obtain the extensive evidence required for disability support claims. This also needs to be considered in the context of individualized funding schemes, and particularly investigated for schemes such as the NDIS, which disproportionately support male participants.

#### The gendered nature of self-advocacy

The central tenet around which individualized funding schemes revolve is one of choice, control, and the empowerment and autonomy of the individual. However, within this framework of choice and control, these schemes also place an exceptionally high emphasis on individuals not only to navigate the system but to act a strong advocates for their own needs [32]. Those better able to advocate for their needs and rights may thus gain disproportionate benefit from a system that strongly relies on self-advocacy [7, 19].

A number of different literatures, including social psychology, management, economics, and health have examined gender differences in self-advocacy and negotiation. As Amanatullah and Morris [39] note, “negotiations are among the most materially consequential of social interactions” (p. 256) and thus understanding the way gender impacts negotiations is vital to ensuring equality and fairness. For example, Bowles et al. [40] found that women’s greater reluctance to initiate negotiations over resources could be explained by the fact that male and female negotiators are treated differently, and particularly that male evaluators penalize women more than men for attempting to negotiate for higher compensation. Women were also more reticent about negotiating for higher compensation when the evaluator was male, which was explained by greater nervousness under that negotiation condition. More recently, Pardal et al. [41] found that men tend to hold implicit and explicit gender stereotypes about face-to-face negotiations and that this can predict lower performance in negotiations for women. Other researchers have argued that women are aware of these implicit and explicit gender stereotypes and that this can affect their behaviour in exerting power and influence when making requests or advocating for themselves [39, 42].

This might be especially so for women with disability from some culturally and linguistically diverse (CALD) communities, where overall participation rates are already very low compared to projected need [43]. CALD and migrant women’s service access can be hindered by difficulty in articulating or asserting their own needs for both cultural and language-related reasons; lack of cultural safety in service provision; or their family situations may reflect a traditional culture of patriarchal control [44, 45].

#### The gendered nature of caregiving

Caregiving is a highly gendered activity that also reproduces gender inequalities [46]. Internationally, women are the main providers of both formal and informal care for children, family members, and those with chronic medical conditions or disabilities (e.g. [46–49]). In Australia, women represent over 70% of primary carers to people with disability and older people. Of those providing primary care to children with disability, nearly 90% are female. Further, 35% of female primary carers have a disability themselves [50].

The caregiving literature has consistently shown that female caregivers experience higher levels of stress and depressive symptoms and are more burdened than male caregivers (e.g. [51–54]. Recently Swinkels et al. [49] examined gender differences in the burden experienced by those caring for partners and found, similar to previous studies, that women feel a greater burden from caregiving than men. Their results suggest this was due to women experiencing more secondary stressors such as having to combine different tasks and financial burdens.

Historically, women with disability have been disregarded as mothers, despite the obvious fact that many women with disability can and do mother [20, 55, 56]. Research suggests women with disability have to work particularly hard and perform significant ‘hidden labour’ to overcome stigma and be perceived as acceptable mothers [57, 58].

Although there has been a significant body of work exploring caregiving through a gender lens, the literature on individualized funding schemes has had less to say about gender impacts for carers under these schemes. Given the undisputed statistics showing the large majority of carers are women it is surprising that studies on individualized funding and caregiving have not focused on gender, or do so only in passing.

Having identified some ways in which women and girls could be disadvantaged through individualized funding schemes, we now describe the methods of the present study.

## Methods

This project was funded by the Disability Innovation Institute UNSW and received ethics clearance through the UNSW HREC (HC200195). We adopted features of inclusive research design in this project, partnering with community organizations and employing a woman with disability to act as peer researcher.

### Interviews

Our two partner organizations – Women with Disabilities ACT and Women with Disabilities Victoria – assisted with participant recruitment. We employed a purposive sampling method [59] to recruit women with disability over the age of 18 who had either applied or considered applying for the NDIS, and ensured that women of different ages and with a wide range of disabilities were represented (see Table 1). Invitations were sent through the partner organizations and women were invited to reply to our peer researcher, who conducted screening, talked participants through the project and its aims, and ascertained accessibility requirements (e.g. needing to look at interview questions beforehand, needing breaks).

We conducted a combination of semi-structured video or audio interviews (depending on participant accessibility needs) between May and October 2020. Two participants chose to have a family member or support worker present, and one interview was conducted with the assistance of an Australian sign language interpreter. Interviews were led by a peer researcher in partnership with an academic researcher, and took between 30 min and 2 h, with most lasting around 1–1.5 h. Participants were assured their contributions would be de-identified, and we gave them the opportunity to choose their own pseudonyms.

All members of the research team and both of the partner organizations had input into the interview questions. These covered participants’ opinions on and experiences with the following:

- Deciding whether to apply for the NDIS
- The application process
- Using the NDIS and communicating with relevant actors
- Whether they were able to get the support they needed
- Caring responsibilities
- Whether there is anything different about being a woman seeking disability support in Australia compared to being a man.

### Analysis

The interviews were transcribed verbatim and then checked for accuracy by a member of the research team. While the overall study was focused on women’s experiences more generally, not just those with a gendered dimension, our aim in this article is to focus on the gendered aspects of women’s experiences. Therefore we have structured our analysis around responses to the question about whether there are differences in men’s and women’s experiences (termed here the ‘key question’). The broader results of the study will be reported elsewhere.

We employed reflexive thematic analysis [60], working deductively to explore further evidence for findings generated by previous research and employing a structural feminist lens informed by Risman’s [61] conception of gender as a social structure.^1^ We did not work from a pre-determined coding frame, instead generating initial codes in NVivo based on participants’ responses to the key question, reflecting on those codes and how they might aggregate into themes, and then returning to the data to refine our coding more systematically according to those themes. While we used responses to the key question as a method of structuring our analysis, in the process of thematic refinement we also drew on data from elsewhere in the interviews, recognising that discussion of gendered barriers would not be limited to one question. In reflexive thematic analysis, themes are “patterns of shared meaning, united by a central concept or idea” [62] (p. 14). This means themes are multifaceted and that data relating to those themes might appear disparate – as is the case, for example, with Theme 1 on self-advocacy and negotiation. However, each piece of data can be conceptually linked to the pattern of meaning that forms the theme. To aid clarity of reporting, we have broken down some themes into sub-themes and explained how each links to the overall pattern.

Having described our approach to data collection and analysis, we now describe our participant sample.

### Participant profile

We encouraged interviewees to self-describe their disability, usually in the context of what supports they received or would like to receive from the NDIS. Some provided broad descriptions (e.g. ‘neurodiverse’), while most provided more specific details. More than a third had multiple disabilities. As our research was informed by the social model of disability [28], we note interviewees’ impairments, where they shared them, in an effort to improve understanding of where trends in NDIS access barriers may occur, and to clarify what types of disability experiences are and are not represented in this research. Examples of impairments participants identified are included in Table 1, along with other demographic details.^2^

**Table 1:** Participant demographics

Characteristic	Details	Number
*Location*	Australian Capital Territory	12
	Victoria	18
*Age*	20s	4
	30s	6
	40s	8
	50s	8
	60s	4
*Impairment types*	*Examples*	*#*
Physical	Spina bifida, arthritis, amputation	17
Neurological	Multiple sclerosis, stroke, acquired brain injury	6
Cognitive	ADHD, autism, other impairments generally classified as intellectual disability or developmental delay	7
Psychosocial	Anxiety, post-traumatic stress disorder (PTSD)	10
Chronic pain and energy impairments	Myalgic encephalomyelitis/chronic fatigue syndrome (ME/CFS)	2
Sensory	Impaired sight, impaired vision	4
*NDIS involvement*	Current plans	24
	Applying	1
	Applying on behalf of children but not self	1
	Considered applying	4
*Race and ethnicity*	Caucasian or undisclosed	25
	Other backgrounds (Aboriginal or Torres Strait Islander, Asian (specific region undisclosed), Pacific Islander, Greek, Italian)	5

## Results

### Are there differences in men’s and women’s experiences?

Of the 30 participants, over two thirds (21) indicated they had perceived differences between men and women’s experiences of accessing disability support, for example describing gendered differences that they had noticed in their own experiences, in the experiences of people they knew, or in the experiences of people with disability more generally. We focus here on results from those who felt women’s experiences did have a gendered dimension.

Participants identified many gendered issues with accessing disability supports. As described above, in analysing and reporting the results we have grouped them into three main (but interconnected) themes. Firstly, *confidence, negotiation and self-advocacy*, with sub-themes of women’s socialisation to be passive and endure; and caring for others before self. Seven participants mentioned this theme explicitly in their response to the question about men’s and women’s different experiences, and we draw on data from 18 participants in discussing the related sub-themes. Secondly, *gendered discrimination in diagnosis and the medical system*, which has implications for disability support access (six participants mentioned this explicitly, and we draw on data from seven participants). Thirdly, *support for and recognition of caring roles*, particularly motherhood but also caring roles beyond the immediate family (four mentioned this explicitly, and we draw on data from nine). *Violence, coercion and safety* also formed a less prominent theme, which it is unfortunately beyond the scope of this article to explore. We provide a map of themes at Appendix 1.

### Theme 1: confidence, negotiation and self-advocacy

Research on gender and self-advocacy or negotiation raises questions about whether women might be disadvantaged in an individualized funding scheme that relies on participant self-advocacy [39, 41].

Over half of the women we interviewed (17–16 of whom were NDIS participants) used adversarial language such as ‘push’, ‘fight’, ‘struggle’, ‘battle’, and ‘argue’ to describe their or others’ interactions with the NDIS, indicating a significant need for self-advocacy. Examples included “I just feel like I’ve had to push so hard” (Peta), “we had the fight” (Dianne), “everything I’ve gotten from the NDIS I have had to push for” (Marjorie), “I had to fight them to get some of the supports that I really really needed” (January), and “so I’m fighting an uphill battle trying to prove that I need things” (Sam).

While some participants felt very able to self-advocate (Daphne described herself as having “more front than Myer”),^3^ more than half of participants mentioned difficulties with self-advocacy, not feeling disabled enough, or putting others before themselves. For example Nellie, an Aboriginal woman with hearing loss, said she had only ever been contacted by men through her NDIS experience, and that she experienced difficulty with those power dynamics:

Nellie: I mean it’s fine, but if you had a white hearing man, then the power is there, and they don’t understand disability on top of that, I feel very uneasy and passive and it’s a little bit like they become the aggressor and I retreat.

While self-advocacy issues were often presented as an individual issue or barrier, some participants connected these themes to wider gendered structures, as discussed below.

#### Women are (or are expected to be) more passive and patient

Several participants commented that differences between men’s and women’s disability experiences stemmed from women being more passive and accepting of circumstances – whether naturally or through learning to ‘do gender’ [64] – or that disability system actors had expectations that women would behave in this way. Jean remarked that men and women can have different disability experiences because “men are much more assertive about getting what they want than women”. Jean had managed to argue for her needs within the NDIS, but had reservations about doing so and acknowledged the trade-offs:

Jean: When I say I'm not assertive, it doesn't come naturally to me. I feel like I have to really work hard to work out why am I not happy, what’s the issue, what can I do about it? And that takes up a lot of energy.

Other participants commented on gendered expectations of endurance and passivity (see e.g. [65]):

Peta: I just think like being a woman …just this idea that you just kind of have to accept to some degree, you just have to put up with it. You know, you're conditioned to care and just put up with shit that comes along or to not speak up if someone offends you.

Melissa: You know, [men with disability] are supported to be empowered, and to be that masculine male dominant figure, whereas I should just be passive and deal with it.

Two participants connected expectations of female passivity to a difference in the responsiveness of disability system actors to women’s needs. In Nellie’s experience:

Nellie: …staff in the [NDIS] offices are more frightened of the violence of a man …I think women are more passive and apologetic and, “sure, I’ll get this for you”, that sort of response. So they take on more of a caring role. So the staff disregard women more than they do men, and men will say, you know, “what’s going on?” and can get more aggressive and more assertive so the staff will respond quicker.

Nellie had helped ‘hundreds’ of people with their NDIS plans and had wondered at first if this observation resulted from her own bias, but then decided “I don’t think this is a bias. I see similarities across different diagnoses, different disabilities and ages of men and women.” Sam perceived a difference in women’s and men’s treatment for a similar reason:

Sam: I think [disability system actors are] a bit more afraid of what the men might do if they don’t get what they want. Whereas we'll go away and think about it, and we'll think there’s no way I can get what I want right now …a man will go, I need this, and it needs to be done now. A man will force it on people more.

#### Backlash for acting against gendered norms

Two others reflected about the gendered double bind of needing to be assertive but being judged by standards of appropriate behaviour for doing so. Family violence survivor Melissa felt a strong need to live independently rather than relying on the care of a man, so she fought hard for what she needed, despite not being comfortable doing so (“…that is really tiring. I’m not a demanding person”). But she experienced backlash for making this effort to be assertive, reflecting “you get labelled as the difficult stubborn disabled woman”. Similarly, Lee had had many negative experiences when self-advocating, and now struggled with it. This had been an issue for her both with her GP when navigating the NDIS application process, and during her initial planning and service utilisation experiences with the NDIS: “I think that …if you’re not a man and you assert yourself, it can be labelled as aggressive or demanding or attention seeking”.

#### Caring for others before self

Several participants related their comments about women’s self-advocacy to their caring roles or being socialized to put others first:

Dianne: I think women are more likely to put themselves second. …because of their caring roles, they often don't put themselves first, whereas men will often put themselves first …And also women are less confident about how to necessarily do that.

This is consistent with the literature on women and caring, which shows that women are expected to be self-sacrificing and to advocate on behalf of others, and do not receive gender-role backlash for it [39, 66]. Dianne was particularly concerned about this in the context of women with disability caring for children with disability:

Dianne: I know of cases where women have been advocating for a child with a disability or an adult child with a disability, and they’ve had to fight all those battles, and then they’re literally too exhausted to fight their own NDIS battle. And if like me, if they've hit a brick wall at some point with what they've asked for, they've just gone oh, I can't do this. So they'll actually go without, rather than continue trying to advocate for themselves.

A concrete example of this in our research was Theresa, who was so engaged with advocating for her children’s disability needs that she had no time to even think about accessing disability services for herself.

Meanwhile, Ruby commented on the shift in view needed to accommodate oneself as needing care instead of (or at the same time as) providing it:

Ruby: I guess we’ve always been, and this is a very sexist thing to say, but we’ve always been the care providers, and so therefore it’s often difficult to become the care recipient, and so that’s also a very different way of looking at yourself.

Skyler also felt that “I’m really great advocating for other people, but not for myself. So there’s a real confidence issue for me in an application to the NDIS.”

This norm of feminine selflessness [66] can go beyond the concrete needs of family members and extend to the needs of unknown others who are more ‘deserving’ of supports. Five participants – including three who had not yet applied for the NDIS – worried that they were “not disabled enough” to apply for supports, or that other people needed it more. As Ruby laughingly reflected: “I thought it was for really disabled people, not for people like me, who are [pauses] really disabled!” Some participants expressed this as a personal barrier of not knowing how to ask for help, but others connected it to gendered expectations of putting others before self, which is consistent with literature suggesting that women are expected to act more pro-socially (i.e. in ways that benefit other people or society as a whole) than men [67].

### Theme 2: gendered discrimination in diagnosis and the medical system

Several participants perceived a difference between men’s and women’s experiences seeking disability support in relation to differences in prevalence and diagnosis of conditions; which conditions are considered eligible for support through the NDIS; and unequal treatment within the medical system.

#### Women’s symptoms dismissed and disbelieved

As discussed above, research has identified a longstanding gender bias within the medical system [68]. Danielle, Sarah, Cyndi, Lee and Marjorie felt that the male-dominated medical profession acted as ‘gatekeepers’ for services, with women finding it harder to get diagnoses, less likely to have their symptoms believed, and less likely to have access granted to services.

Lee: I'm just thinking, just accessing medical help, disability help, I really often feel like I'm not being heard because I'm a woman, or I'm not being believed.

Danielle noted that about three quarters of people with ME/CFS are women, and that this had an impact on how the condition was viewed by the disability support system:

Danielle: So I think it's really complex, but I think the way that women's symptoms and illnesses that are associated with women in particular get treated by the medical profession has a big impact on who has access to support and who doesn't.

While some of these examples were set in health services, they demonstrate women’s repeated experiences of being discredited, often in contrast to the credibility attributed to men.

#### Disparities in conditions most likely to receive funding

Several participants perceived gendered inequalities in the types of conditions most likely to receive NDIS funding, with those predominant in men more likely to receive funding and those predominant in women less likely. Marjorie had not personally experienced difficulty accessing the NDIS, but had seen differences in how others were able to access the scheme:

Marjorie: [I observed] men who were talking about how easy it was for them to access the NDIS, and then groups of women who were saying why am I getting shot down constantly? Why are they wanting more and more information about conditions that they should be aware about? Why can I get no help for endometriosis, which is completely debilitating …you know this kind of inequality was so striking.

January accessed NDIS support for her ME/CFS, and noted that this condition is more prevalent in women, and that she knew many people whose impairments were as severe as her own and yet could not get access, or were having their services reduced. She reflected:

January: There's more men than women diagnosed as autistic. Although, whether that reflects the reality or that's a label mistake, I understand people aren’t sure, but there's also more young men than young women that have spinal cord injuries and stuff. And all of these things men are more likely to be diagnosed with, they're all in List B.^4^ They're much easier to get into NDIS with. The things that women are more likely to be diagnosed with, which are overwhelmingly autoimmune conditions, chronic illness-based conditions, these are the things the NDIS spends a really enormous amount of energy trying to convince you that you can't use NDIS for.

Joanne’s story underscores some of the difficulties with being diagnosed with autism as a woman or girl: she had recently been diagnosed with ‘level two’ autism (which is "pretty much always" granted access to the NDIS) and had found applying for the scheme to be relatively straightforward. Since diagnosis her life had improved considerably, but she had endured years of previous misdiagnoses and had only reached this point through significant self-advocacy:

Joanne: I very much had to seek out that diagnosis and discover it for myself. At no point over the 12 years of psychotherapy and psychiatrist appointments did anyone suggest autism to me, because I’m a woman.

As a behavioural support service provider for NDIS participants, she also noticed a lack of women and girls with ‘low’ support needs, “because it’s those lower support needs women and girls that are flying under the radar” due to underdiagnosis.

#### Barriers to entry

Cyndi and Danielle would both have liked to access services through the NDIS, but both felt that it would be very difficult to do this given their primary diagnoses with conditions more prevalent in women and their observations of how the NDIS treated women with these conditions. Cyndi told us “EDS [Ehlers-Danlos Syndrome] disproportionately affects women and like all things, is therefore underdiagnosed, under-researched, so on and so forth”. Despite significant impacts on her life and despite feeling that early intervention was important for later functionality, she had decided not to apply for NDIS support. This was due to seeing so many people "knocked back", even with significant support from “doctors who know how to fill out all of the forms and all the sort of crap you have to do to get the NDIA to listen …I don’t know anyone with EDS as their primary diagnosis who has gotten onto the scheme.” Likewise Danielle had observed through online support groups for her condition that “maybe 10 per cent of people who need it are getting it”, primarily those who were bedridden or housebound.

### Theme 3: support for and recognition of caring roles

The third major theme in participants’ perceptions of gendered barriers to the NDIS related to motherhood, childcare and other caring responsibilities. This has been noted in the literature as a significant issue for women with disabilities, who are often not perceived as competent mothers, or have their caring roles disregarded or unsupported – some while simultaneously experiencing ‘overservicing’ related to surveillance and child protection [20].

As noted above, several participants had caring responsibilities for children or wanted to be supported to have more of a caring role. Several participants felt their caring roles or family relationships were not adequately recognized by the NDIS or by society more generally:

Cat: I don’t think there’s any sort of acknowledgement often of people who do have disabilities who [also] have caring responsibilities. …as a person with a disability you must be cared for, you are the recipient.

Melissa: …my motherhood gets completely thrown out the window. …they don’t see me as a mother, like other women.

Theresa felt that as a mother of several children with disability, she was expected to know how to access care for them. While she had no time to think about support for her own disability, she strongly wished for support to help her access the NDIS and other services on behalf of her children:

Theresa: I don't know whether it would be an advocate or what, that just kind of helps me navigate the process for the kids. So it's almost- I joke that I need a wife, but someone to help me that I can be honest with, and for them not to threaten child protection or tell me I've got bad DNA or whatever.

This quote illustrates many complexities of mothering in the context of disability: difficulty with service access, fear of judgement, fear of having children removed, and a humorous acknowledgement that women bear the brunt of reproductive labour and organizing for disability services (“I need a wife”).

Child removal and fear of child protection was also a concern for several other participants, who felt that women with disability can attract the wrong kind of attention from government services if they admit to needing help or if their disability is not adequately supported.

#### Insufficient recognition of mothering role by the NDIS

For some people, insufficient recognition of caring roles was about what children were expected to do to support parents with disability, in a reversal of normal parent-child relationships:

Dianne: I know why they say that family members can't be paid carers, but there are times when I wish you could. …You know, your daughter doesn't normally take mum to the bathroom. So that's why I think it's a bit unfair that you can't recognize when they're actually doing tasks that are above and beyond a normal family role.

Likewise Melissa felt that in expecting her teenage son to provide intimate care such as showering, NDIS planners were not supporting her to be a mother to him:

Melissa: …putting the responsibility to look after me onto my kids, because they’re grown up. And not understanding why that would be a problem, and how that impacts a relationship between a mother and a teenager.

Jackie and Lily also felt that the NDIS did not provide sufficient recognition of their mothering roles, Jackie connecting this to wider discrimination against parents with intellectual disability:

Jackie: The NDIS came to self-advocacy groups and said what’s missing, and I said parents being able to keep their children with the support they need.

Jackie’s daughter was not in her care but she wanted support to have a greater role in her daughter’s life. However, she found it difficult to access parenting programs because they were not aimed at parents with intellectual disability (using ‘jargon’ that is difficult to understand) and most were not available to parents who did not have a direct caring role.

Lily reported “absolutely zero consideration” of her full-time caring role for her adult son with “severe and complex disabilities”, although both had NDIS plans. While they had support workers during the day, Lily was required to provide her son’s care at night, but she had been told not to mention him in her NDIS plan:

Lily: They just say no, we can’t include [son] in yours, so they keep it totally separate. With [son]- so they don't give any consideration of- even having any respite. I don’t have any respite.

Later, she commented:

Lily: …it’s a real concern that they keep us so separate when we actually live together, and I’ve never been in any other service, all of our lives, that didn’t give the parent consideration, until the NDIS.

### Caring beyond the immediate family

Nellie, an Aboriginal woman, described extensive caring and decision-making responsibilities as a senior person in her family and an Elder in her community. She cared for her father, siblings, nieces and nephews, grandchildren, and other family members: “It’s a very different role, I guess, Indigenous-wise, than just a mainstream cultural role of caring.” As a deaf woman, Nellie needed access to interpreting support to undertake these responsibilities, but had been told she could only get 75 h per year – which for her “will run out in two weeks”. Living in a regional town, there were also not many sufficiently qualified and trained interpreters in her area. She felt that the NDIS did not understand her need for services to provide care to her family and community:

Nellie: I don’t believe that the NDIS themselves really have any sort of clue of what it means when you’re talking about the Aboriginal context and you’re talking about women within the Aboriginal context.

Cat too had caring responsibilities for her sister, who had an intellectual disability, but had been ‘rejected’ for a carer payment through welfare agency Centrelink. She did not feel her caring role was recognized by the NDIS, but felt this was reflective of a wider societal inability to understand that people with disability can be carers as well as cared for:

Cat: So I’m a support for her I guess in terms of …social emotional support I guess, not so much physical. But I certainly help her with budgeting and all that kind of stuff, and keeping things under control. But they don’t recognize that type of caring, that’s of no consequence apparently.

## Discussion

Previous research on the NDIS and on other individualized funding schemes has warned that such schemes can widen inequalities along lines of discrimination and disadvantage that already exist in the wider society. It is clear from this research that some women accessing the NDIS experience gendered issues that cause or exacerbate barriers to support. While gender inequalities may still be present in other systems of disability support such as block funding of providers, there are specific attributes of individualized funding approaches that may act to increase these inequalities. Individualized funding schemes require unprecedented emphasis on individual advocacy and administration skills; skills which are not equally distributed across the population [18, 25]. We found that women may be disadvantaged through systems such as the NDIS that are complex to navigate, rely on self-advocacy, and require considerable work from the individual (or their family members). This is consistent with a previous evaluation of the NDIS, which found that male participants were less likely to experience unmet support demand than female participants [69].

Not all the women we talked to perceived gendered barriers to disability support through the NDIS – several had had very positive experiences with the NDIS, or did not perceive gendered differences to be a factor in any barriers they might have experienced. However, *most* of our participants did perceive differences, either in their personal experiences compared to those of men, or in the experiences of women with disability compared to men with disability more generally. Some perceived a barrier related to self-advocacy, feeling that women may be at a disadvantage in individualized funding schemes where those who are effective self-advocates are more likely to gain greater benefit. Several felt that self-advocacy might come more naturally to men or that NDIS system actors might receive men who prioritize their own needs more positively than women. In line with Risman’s [61] theory of gender as a multi-level social structure, this is consistent with gender processes affecting women’s experiences at the individual level (where their gendered selves and identities may be shaped by socialisation and internalisation of norms), at the interactional level (where women may experience backlash when coming up against expectations of traditional feminine behaviour, or may modify their behaviour in the anticipation of backlash), and even at the institutional level (if there is a culture of responding more positively to men’s assertiveness than women’s, as several participants suggested).

These findings suggest that the social costs for women in behaving in a way that goes against gender norms, such as being assertive or self-advocating, may contribute to gender inequalities in individualized funding schemes. It is possible that women may be less likely than men to try and advocate assertively for their needs with service providers or in planning meetings as they worry they will be perceived in a negative way or disliked, and this could lead to them missing out on funding or services. In a study on service users’ perceptions of having choice and control in the NDIS, Mavromaras et al. [69] found that participants who were less able to articulate their support needs experienced less choice and control over their supports. On the other hand, if they do act assertively, they may experience negative consequences. Warr et al. [32] explored participants’ experiences of having choice and control in the NDIS and found that some participants feared that they could be denied services because they were perceived as ‘too difficult’ by staff, who could choose their clients when there was high demand for services. Malbon et al. [7] also argued that while choice and control in the NDIS is intended to sit with participants, the creation of a new services marketplace means that service providers and individual workers will also be able to exercise greater choice in the clients they want to work with. This contrasts with more traditional block-funded service delivery models where service providers are not able to pick and choose their clients.

Some participants felt that a historically male-dominated and gender biased medical system had shaped the disability support system and was keeping them or other women from accessing the supports they needed. This was particularly perceived to be a problem in relation to ‘invisible disabilities’ that are more prevalent in women [70], and yet form only a small part of the NDIS. Also, as discussed in the literature and exemplified by our late-diagnosed participant Joanne, there are issues with the late diagnosis and under-diagnosis of autism in women and girls [14]. Autism is the most common primary disability of NDIS participants (32%), and there are nearly three times more male than female participants with autism [9]. Male overrepresentation in the scheme has largely been attributed to greater prevalence of autism (as well as intellectual disability and developmental delay) in boys [8].

Finally, several participants perceived inequalities and barriers for women related to parenting or caring responsibilities. While of course men with disability are also parents, barriers related to parenting and caring support are more significant for women because gender structures place mothers in a more significant caring role to their children [20] – for example, mothers with disability are more likely to have care of children and other family members than fathers with disability [50]. The early implementation of the NDIS was criticized for not including parenting as a ‘disability-related’ support need [71], however disability advocacy was successful, and this was rectified with the full implementation. Several women we interviewed felt that their caring responsibilities were not adequately recognized or supported by the NDIS, or felt an ambivalence about asking for the help they required because they worried that in needing help, they would be perceived as unfit mothers. This echoes research about the paradox of mothering with disability, where scholars have argued that mothers face both *underservicing*, in the sense of not being recognized in their caring roles and not receiving appropriate supports to undertake those roles, at the same time as *overservicing*, in the form of surveillance by social welfare agencies and the threat of child removal if they are judged as inadequate ([20]; see [72] for evidence that this still occurs in Australia).

In Aboriginal communities (which have overrepresentation of people with disability but underrepresentation of participants in the NDIS), women Elders such as Nellie have significant caring and decision-making responsibilities. Nellie’s experiences in particular illustrate that intersectionality – the unique experiences of people located at the intersections of oppressive social structures [30] – must be taken into account when considering women’s experiences of accessing the NDIS. It became clear that some of her experiences could only be understood when considering her position as a woman and as an Aboriginal Elder, and the barrier of not being provided with enough interpreting hours to fulfil the associated caring responsibilities for extended family and community. Added to this was her regional location and the lack of adequately skilled interpreters in her area (for example, she could not use a male interpreter to discuss women’s business), compounded by a lack of understanding from the NDIA staff she interacted with about any of these issues.

### Implications for practice

As the NDIS aims to achieve choice and control for *all* Australians with disabilities, there is a case for all levels of service design and provision to address gender inequality, and certainly not entrench it (which our findings suggest may be occurring). As noted, the NDIS has experienced a range of implementation challenges and has been subject to considerable political debate and pressure, particularly around its cost [73–75]. Amongst the criticisms of the main implementation agency, the NDIA, has been a lack of understanding of disability [25]. Arguably, this lack of understanding has combined with cost cutting pressures to create less than ideal planning experiences and outcomes for participants, including those described in this research.

Specifically with relation to gendered experiences, previous research in policy implementation and administration has shown that without a concerted focus on gender, and structural changes, there is a tendency to revert to gender-biased ways of working [76, 77]. This work suggests that training NDIA staff in concepts such as unconscious bias alone is unlikely to create the requisite systemic change. Rather, this training needs to be accompanied by a commitment to gender equality, including targets and thorough evaluation of women’s experiences in order to improve practice [77].

Other strategies to address gender inequalities within the NDIS include better integration between the NDIS/NDIA and existing women’s services – for example, building a system interface between the NDIS and critical women’s services such as specialist domestic and family violence services, sexual and reproductive health services, and parenting and carer support services. This could be underpinned by resourced policy and governance links between key strategies such as the National Disability Strategy and the National Plan to Address Violence Against Women and their Children.

Concerningly there has also been a defunding of disability advocacy organizations since the launch of the NDIS, consistent with the individualized logic of self-advocacy [78]. The gendered experiences of women explored in this study demonstrate the need for sustained funding for women’s disability organizations.

### Limitations and future directions

This is an exploratory study intended to uncover and describe gendered issues with individualized funding, as no research has done this previously. With a sample size of 30, it is not intended to be a comprehensive exploration of gendered barriers. Further, due to recruitment through advocacy organizations, the sample skewed white and well-educated, so future research focused more on the inclusion of women across more diverse racial and socioeconomic groups will be important. Recent research by Carey and colleagues [79]  indicates that the experiences of culturally and linguistically diverse women will be particularly important to explore. Another helpful expansion to this line of research would be the inclusion of men to allow for comparisons between the experiences of men and boys and women and girls, perhaps using a survey methodology to reach a larger group of participants.

## Conclusion

Our research provides the first in-depth look at women’s experiences of individualized or personalized funding schemes, employing a specifically gendered lens. While one of the central tenets of personalization is that it better accounts for differences in need, background and culture [80], our research adds to a growing body of work that indicates that these schemes may in fact entrench or widen existing inequalities. It’s important to note this is not a fait accompli – there is much that can be done through considered design and implementation to overcome these issues [19]. In the context of gender, existing research indicates that a gender plan, with attached accountabilities, is key to overcoming the inequalities described in this paper. This stretches from explicit inclusion of women with disabilities in design, through to good accountability structures to ensure efforts to address gendered inequalities are fully evaluated and refined over time [81].

## Data Availability

The data that support the findings of this qualitative study are not publicly available due to ethics requirements.

## Footnotes

1 ‘Deductive’ in this context means acknowledging that it is not possible to approach analysis in a theoretical vacuum, and drawing on the concept of gender as a social structure as a lens through which to code and interpret data [62].

2 Categories largely based on those used by CRE-DH [63]. We use ‘psychosocial’ rather than ‘psychological’ because this accords with NDIS terminology. ‘Energy impairments’ was used by our interviewees for ME/CFS and similar conditions.

3 An Australian idiom referring to a department store with significant street-frontage.

4 At the time the research was conducted, NDIS eligibility lists were used to determine if someone would be very likely to gain access to the NDIS (List A and D) or may gain access if more evidence about functional capacity were provided (List B). Conditions that were not on any of the lists were unlikely to be funded as they were not considered ‘permanent’.

## References

[1] Carey G, Malbon E, Olney S, Reeders D (2018). The personalisation agenda: the case of the Australian National Disability Insurance Scheme. Int Rev Sociol.

[2] Bornat J, Leece J (2006). Developments in direct payments.

[3] Fleming P, McGilloway S, Hernon M, Furlong M, O’Doherty S, Keogh F (2019). Individualized funding interventions to improve health and social care outcomes for people with a disability: a mixed-methods systematic review. Campbell Syst Rev.

[4] Williams I, Dickinson H (2016). Going it alone or playing to the crowd? A critique of individual budgets and the personalisation of health Care in the English National Health Service. Aust J Public Adm.

[5] Gadsby EW. Personal budgets and health: a review of the evidence. London (UK): PRUComm. 2013. http://blogs.lshtm.ac.uk/prucomm/files/2013/04/Personal-Budgets-review-of-evidence_FINAL-REPORT.pdf. Accessed 17 Nov 2015.

[6] Gash T, Panchamia N, Sims S, Hotson L (2013). Making public service markets work.

[7] Malbon E, Carey G, Meltzer A (2019). Personalisation schemes in social care: are they growing social and health inequalities?. BMC Public Health.

[8] NDIS (2019). Analysis of participants by gender.

[9] NDIS (2021). NDIS quarterly report to disability ministers.

[10] Soldatic K, van Toorn G, Dowse L, Muir K (2014). Intellectual disability and complex intersections: marginalisation under the National Disability Insurance Scheme. Res Pract Intellect Dev Disabil.

[11] United Nations. Convention on the rights of persons with disability. 2006. https://www.un.org/development/desa/disabilities/convention-on-the-rights-of-persons-with-disabilities/convention-on-the-rights-of-persons-with-disabilities-2.html.

[12] Mavromaras K, Moskos M, Mahuteau S (2016). Evaluation of the NDIS.

[13] Women With Disabilities Australia, Women with disabilities Australian Capital Territory, Women With Disabilities South Australia, Women With Disabilities Victoria. Regarding Gender Inequality in the NDIS. 2018. https://www.dvnsw.org.au/wp-content/uploads/2018/11/Joint_Letter_CEO_NDIA_Final.pdf. Accessed 2 Jul 2019.

[14] Gould J (2017). Towards understanding the under-recognition of girls and women on the autism spectrum. Autism..

[15] Desai MK, Brinton RD. Autoimmune disease in women: endocrine transition and risk across the lifespan. Front Endocrinol. 2019;10. 10.3389/fendo.2019.00265. 10.3389/fendo.2019.00265 PMC6501433 31110493

[16] Briones-Vozmediano E, Öhman A, Goicolea I, Vives-Cases C (2018). “The complaining women”: health professionals’ perceptions on patients with fibromyalgia in Spain. Disabil Rehabil.

[17] Arout CA, Sofuoglu M, Bastian LA, Rosenheck RA (2018). Gender differences in the prevalence of fibromyalgia and in concomitant medical and psychiatric disorders: a National Veterans Health Administration Study. J Women's Health (Larchmt).

[18] Babcock L, Laschever S. Women Don’t ask: negotiation and the gender divide. Princeton: Princeton University Press; 2003.

[19] Carey G, Crammond B, Malbon E (2019). Personalisation schemes in social care and inequality: review of the evidence and early theorising. Int J Equity Health.

[20] Malacrida C. Mothering and disability: implications for theory and practice. In: Watson N, editor. Routledge Handbook of Disability Studies. 2012. p. 390–401.

[21] Thill C (2015). Listening for policy change: how the voices of disabled people shaped Australia’s National Disability Insurance Scheme. Disabil Soc..

[22] Productivity Commission (2011). Disability care and support: productivity commission inquiry report.

[23] Collings S, Dew A, Dowse L (2016). Support planning with people with intellectual disability and complex support needs in the Australian National Disability Insurance Scheme. J Intellect Dev Disabil.

[24] ANAO (2016). National Disability Insurance Scheme - Management of Transition of the disability services market.

[25] Hansard, Commonwealth Government of Australia. Joint Standing Committee on the National Disability Insurance Scheme: Market readiness for provision of services under the National Disability Insurance Scheme. Hansard, Commonwealth Government of Australia; 2018.

[26] Tune D (2019). Review of the National Disability Insurance act 2013: removing red tape and implementing the NDIS participant service guarantee.

[27] Yates S (2020). Gender, context and constraint: framing family violence in Victoria. Womens Stud Int Forum.

[28] Berger RJ, Lorenz LS. Disability and Qualitative Inquiry: Methods for Rethinking an Ableist World. London: Routledge; 2016.

[29] Traustadóttir R (2006). Disability and gender: introduction to the special issue. Scand J Disabil Res.

[30] Weldon SL, Goertz G, Mazur A (2008). Intersectionality. Politics, gender, and concepts: theory and methodology.

[31] Carey G, Malbon E, Blackwell J. Administering inequality? The National Disability Insurance Scheme and administrative burdens on individuals. Aust J Public Adm. 2021. 10.1111/1467-8500.12508.

[32] Warr D, Dickinson H, Olney S, Karanikolas A, Kasidis V, Katsikis G (2017). Choice, control and the NDIS service users’ perspectives on having choice and control in the new National Disability Insurance Scheme.

[33] Jackson G. Pain and prejudice. Piatkus; 2019. https://www.booktopia.com.au/pain-and-prejudice-gabrielle-jackson/book/9781760529093.html. Accessed 13 May 2021.

[34] Criado PC (2019). Invisible women: data Bias in a world designed for men.

[35] Wood-Downie H, Wong B, Kovshoff H, Mandy W, Hull L, Hadwin JA. Sex/gender differences in camouflaging in children and adolescents with autism. J Autism Dev Disord. 2020. 10.1007/s10803-020-04615-z. 10.1007/s10803-020-04615-z PMC7985051 32691191

[36] Zener D (2019). Journey to diagnosis for women with autism. Adv Autism.

[37] Samulowitz A, Gremyr I, Eriksson E, Hensing G. “Brave men” and “emotional women”: a theory-guided literature review on gender bias in health care and gendered norms towards patients with chronic pain. Pain Res Manage 2018;2018:e6358624. doi:https://doi.org/10.1155/2018/6358624. 10.1155/2018/6358624 PMC5845507 29682130

[38] Mauvais-Jarvis F, Merz NB, Barnes PJ, Brinton RD, Carrero J-J, DeMeo DL (2020). Sex and gender: modifiers of health, disease, and medicine. Lancet.

[39] Amanatullah ET, Morris MW (2010). Negotiating gender roles: gender differences in assertive negotiating are mediated by women’s fear of backlash and attenuated when negotiating on behalf of others. J Pers Soc Psychol.

[40] Bowles HR, Babcock L, Lai L (2007). Social incentives for gender differences in the propensity to initiate negotiations: sometimes it does hurt to ask. Organ Behav Hum Decis Process.

[41] Pardal V, Alger M, Latu I (2020). Implicit and explicit gender stereotypes at the bargaining table: male counterparts’ stereotypes predict Women’s lower performance in dyadic face-to-face negotiations. Sex Roles New York.

[42] Amanatullah ET, Tinsley CH (2013). Punishing female negotiators for asserting too much…or not enough: exploring why advocacy moderates backlash against assertive female negotiators. Organ Behav Hum Decis Process.

[43] Young E. Percentage of culturally diverse NDIS participants still more than halfway off 2018 projections. SBS News 2021. https://www.sbs.com.au/news/percentage-of-culturally-diverse-ndis-participants-still-more-than-halfway-off-2018-projections/21bc7d77-e7b9-4b3a-87ca-b728b33c5d8e. Accessed 10 Sep 2021.

[44] Mengesha Z, Dune T, Perz J (2016). Culturally and linguistically diverse women’s views and experiences of accessing sexual and reproductive health care in Australia: a systematic review. Sex Health.

[45] Casimiro S, Hancock P, Northcote J (2007). Isolation and insecurity: resettlement issues among Muslim refugee women in Perth, Western Australia. Aust J Soc Issues.

[46] Adams V (2010). Scoping the Australian Care Economy A Gender Equity Perspective.

[47] Revenson TA, Griva K, Luszczynska A, Morrison V, Panagopoulou E, Vilchinsky N (2016). Gender and caregiving: the costs of caregiving for women. Caregiving in the illness context.

[48] Sharma N, Chakrabarti S, Grover S (2016). Gender differences in caregiving among family - caregivers of people with mental illnesses. World J Psychiatry.

[49] Swinkels J (2019). Tilburg T van, Verbakel E, Broese van Groenou M. explaining the gender gap in the caregiving burden of partner caregivers. J Gerontol B Psychol Sci Soc Sci.

[50] ABS. Disability, Ageing and Carers, Australia: Summary of Findings, 2018. ABS; 2019. https://www.abs.gov.au/statistics/health/disability/disability-ageing-and-carers-australia-summary-findings/latest-release. Accessed 9 Jun 2021.

[51] Caputo J, Pavalko EK, Hardy MA (2016). The long-term effects of caregiving on Women’s health and mortality. J Marriage Fam.

[52] Marks NF, Lambert JD, Choi H (2002). Transitions to caregiving, gender, and psychological well-being: a prospective U.S. national study. J Marriage Family.

[53] McDonnell E, Ryan A (2013). Male caregiving in dementia: a review and commentary. Dementia (London).

[54] Pinquart M, Sörensen S (2003). Associations of stressors and uplifts of caregiving with caregiver burden and depressive mood: a meta-analysis. J Gerontol B Psychol Sci Soc Sci..

[55] Vaidya S (2015). Women with disability and reproductive rights: deconstructing discourses. Social Change.

[56] Malacrida C (2009). Performing motherhood in a disablist world: dilemmas of motherhood, femininity and disability. Int J Qual Stud Educ.

[57] Frederick A (2017). Visibility, respectability, and disengagement: the everyday resistance of mothers with disabilities. Soc Sci Med.

[58] Shpigelman C-N (2015). How to support the needs of mothers with physical disabilities?. Disabil Rehabil.

[59] Tong A, Sainsbury P, Craig J (2007). Consolidated criteria for reporting qualitative research (COREQ): a 32-item checklist for interviews and focus groups. Int J Qual Health Care.

[60] Braun V, Clarke V (2006). Using thematic analysis in psychology. Qual Res Psychol.

[61] Risman BJ (2004). Gender as a social structure: theory wrestling with activism. Gend Soc.

[62] Braun V, Clarke V. One size fits all? What counts as quality practice in (reflexive) thematic analysis? Qual Res Psychol 2020;0:1–25.

[63] Centre of Research Excellence in Disability and Health. Violence against people with disability in Australia. Fact Sheet 1. Centre of Research Excellence in Disability and Health (CRE-DH); 2020. https://credh.org.au/nature-and-extent-of-violence/. Accessed 11 May 2020.

[64] West C, Zimmerman DH (1987). Doing Gender. Gend Soc.

[65] Gilbert PR (2002). Discourses of female violence and societal gender stereotypes. Violence Against Women.

[66] Wade ME (2001). Women and salary negotiation: the costs of self-advocacy. Psychol Women Q.

[67] Heilman ME, Chen JJ (2005). Same behavior, different consequences: reactions to Men’s and Women’s altruistic citizenship behavior. J Appl Psychol.

[68] Hamberg K (2008). Gender Bias in Medicine. Women's Health (Lond Engl).

[69] Mavromaras K, Moskos M, Mahuteau S, Iskerwood L (2018). Evaluation of the NDIS: final report.

[70] Hirschmann NJ (2012). Disability as a new frontier for feminist intersectionality research. Polit Gend.

[71] Collings S, Strnadová I, Loblinzk J, Danker J (2020). Benefits and limits of peer support for mothers with intellectual disability affected by domestic violence and child protection. Disabil Soc.

[72] Carter B (2015). Rebuilding the village: supporting families where a parent has a disability : report 2 : child protection.

[73] Joint Standing Committee on the National Disability Insurance. Independent Assessments. Commonwealth Government of Australia; 2021.

[74] Morton R. Families’ NDIS support slashed in crackdown. The Australian 2017.

[75] Nevile A, Kay A, Carey G. Value choices in a mixed economy of care: how politics shapes the implementation of complex social policies. Soc Policy Adm. 2018. 10.1111/spol.12391.

[76] Williamson S, Colley L (2018). Gender in the Australian public service: doing, undoing, redoing or done?: gender in the Australian public service. Aust J Public Adm.

[77] Williamson S, Foley M (2018). Unconscious Bias training: the ‘silver bullet’ for gender equity?: unconscious Bias training. Aust J Public Adm.

[78] Carey G, Weier M, Duff G, Dickinson H. How is the disability sector faring? A report from National Disability Services. CSI UNSW; 2020.

[79] Carey G, Malbon E, Blackwell J. Administering inequality? The National Disability Insurance Scheme and administrative burdens on individuals. Australian Journal of Public Administration. 2021. 10.1111/1467-8500.12508.

[80] Carey G, Crammond B. A glossary of policy frameworks: the many forms of “universalism” and policy “targeting.” J Epidemiol Community Health 2017;71:303. 10.1136/jech-2014-204311 25294894

[81] Carey G, Crammond B (2015). What works in joined-up government? An evidence synthesis. Int J Public Adm.

